# Sialic Acid and Fucose Residues on the SARS-CoV-2 Receptor-Binding Domain Modulate IgG Antibody Reactivity

**DOI:** 10.1021/acsinfecdis.2c00155

**Published:** 2022-08-18

**Authors:** Ebba Samuelsson, Ekaterina Mirgorodskaya, Kristina Nyström, Malin Bäckström, Jan-Åke Liljeqvist, Rickard Nordén

**Affiliations:** † Department of Infectious Diseases, Institute of Biomedicine, Sahlgrenska Academy, University of Gothenburg, Gothenburg 413 46, Sweden; ‡ Proteomics Core Facility, Sahlgrenska Academy, University of Gothenburg, Gothenburg 413 90, Sweden; § Mammalian Protein Expression Core Facility, Sahlgrenska Academy, University of Gothenburg, Gothenburg 413 90, Sweden; ∥ Department of Clinical Microbiology, Region Västra Götaland, Sahlgrenska University Hospital, Gothenburg 413 45, Sweden

**Keywords:** antibody reactivity, glycoepitope, glycoproteomics, mass spectrometry, receptor binding domain, SARS-CoV-2

## Abstract

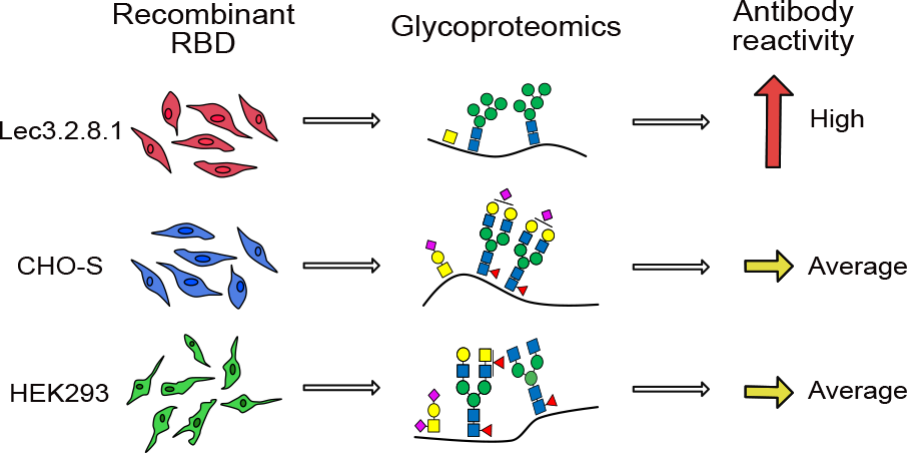

The receptor-binding domain (RBD) of the SARS-CoV-2 spike protein is a conserved domain and a target for neutralizing antibodies. We defined the carbohydrate content of the recombinant RBD produced in different mammalian cells. We found a higher degree of complex-type N-linked glycans, with less sialylation and more fucosylation, when the RBD was produced in human embryonic kidney cells compared to the same protein produced in Chinese hamster ovary cells. The carbohydrates on the RBD proteins were enzymatically modulated, and the effect on antibody reactivity was evaluated with serum samples from SARS-CoV-2 positive patients. Removal of all carbohydrates diminished antibody reactivity, while removal of only sialic acids or terminal fucoses improved the reactivity. The RBD produced in Lec3.2.8.1-cells, which generate carbohydrate structures devoid of sialic acids and with reduced fucose content, exhibited enhanced antibody reactivity, verifying the importance of these specific monosaccharides. The results can be of importance for the design of future vaccine candidates, indicating that it is possible to enhance the immunogenicity of recombinant viral proteins.

## Introduction

The adaptive immune response to SARS-COV-2 depends on T-cells that direct the immune responses and contribute to killing of infected cells and on antibody-producing B-cells.^1^ Seroconversion has been detected in 93–99% of patients with diagnosed SARS-CoV-2 infection, with disease severity correlating with antibody titres.^2−4^ Neutralizing antibodies (NAb) are a key component in the response toward viruses, and an important aspect after immunization is whether the generated antibodies possess neutralizing capabilities.^5^ In the case of SARS-CoV-2, many neutralizing antibodies recognize the receptor-binding motif (RBM) within the receptor-binding domain (RBD) of the spike (S) protein^6^ and execute their neutralizing capacity by sterically hindering viral binding to the angiotensin converting enzyme 2 (ACE2) receptor^7^ or by keeping the RBD in its “down-conformation”.^6^ However, there are reports about neutralizing antibodies targeting epitopes also outside the RBM, such as 47D11, which binds to the conserved core of the RBD,^8^ or S309, which recognizes a conserved epitope involving interactions with the fucose and other glycan moieties of the N343-glycan within the RBD.^9^ Neutralizing antibodies have also been found to target the N-terminal domain (NTD) of the S protein. For example, the NAb 4A8 targets residues within the NTD, including the N147-glycosite, and neutralizes possibly by inhibiting conformational changes of the S protein.^10^ Thus, the glycosylation profile of the S protein appears to be important for antibody recognition and neutralization.

The S protein is inserted in the viral envelope as a trimer, forming the characteristic “spikes” protruding from the viral surface. The S protein is cleaved by host proteases to form S1 and S2. The S1 domain contains the RBM and mediates binding to the ACE2 receptor, while fusion with the host cell membrane is mediated by S2.^11−13^ The S protein ectodomain contains 22 consensus sites for N-linked glycosylation (Asn-X-Ser/Thr where X is any amino acid except Pro). Most of the sites have been reported as glycosylated, carrying complex and high-mannose glycans for recombinant S proteins, expressed in cell culture.^14−17^ Although most consensus sites for N-linked glycosylation in the S protein appear to be occupied, the composition and structure of glycans at respective sites appear highly variable.^15,16^ The O-linked glycosylation pattern of the S protein is not entirely established, although the presence of several O-linked glycans has been identified within the RBD.^14,17−19^

The vaccines against SARS-CoV-2 induce antibodies that after immunization target specific domains of the S protein. The vector-based DNA vaccines and mRNA vaccines utilize the human glycosylation profile on the produced protein, while the glycosylation profile of protein-based subunit vaccines is dependent on the cell type used for production.^20^ It has been shown that it is possible to alter the glycan content of the S protein without affecting the serological properties,^21^ and by truncating the glycans, it was possible to enhance protection in an animal model.^22^ However, the glycan content is also important for correct folding of the S protein and may influence the antigen stability and could therefore indirectly affect the distribution and presentation of the antigen.^23−25^ Thus, the glycosylation profile of the S protein may directly affect the antibody recognition by shielding specific epitopes, or indirectly by altering the architecture of the protein, leading to variability in the effectivity of a potential vaccine candidate.^26,27^

In this work, we have characterized the glycan content of a recombinant RBD protein expressed in three different mammalian cell lines and showed a diverse glycan composition at each site. The N- and O-linked glycans were stepwise modulated using enzymatic degradation. Serum samples from patients previously infected with SARS-CoV-2 were used to assess the impact of glycan composition on antibody reactivity. A glycan hot spot within the RBD was found to be essential for antibody reactivity. In addition, modulation of the glycan content revealed specific monosaccharides that were able to enhance the antibody reactivity.

## Results

### Glycosylation Pattern of the Recombinant RBD Produced in CHO-S and HEK293F Cells

**Figure 1:**
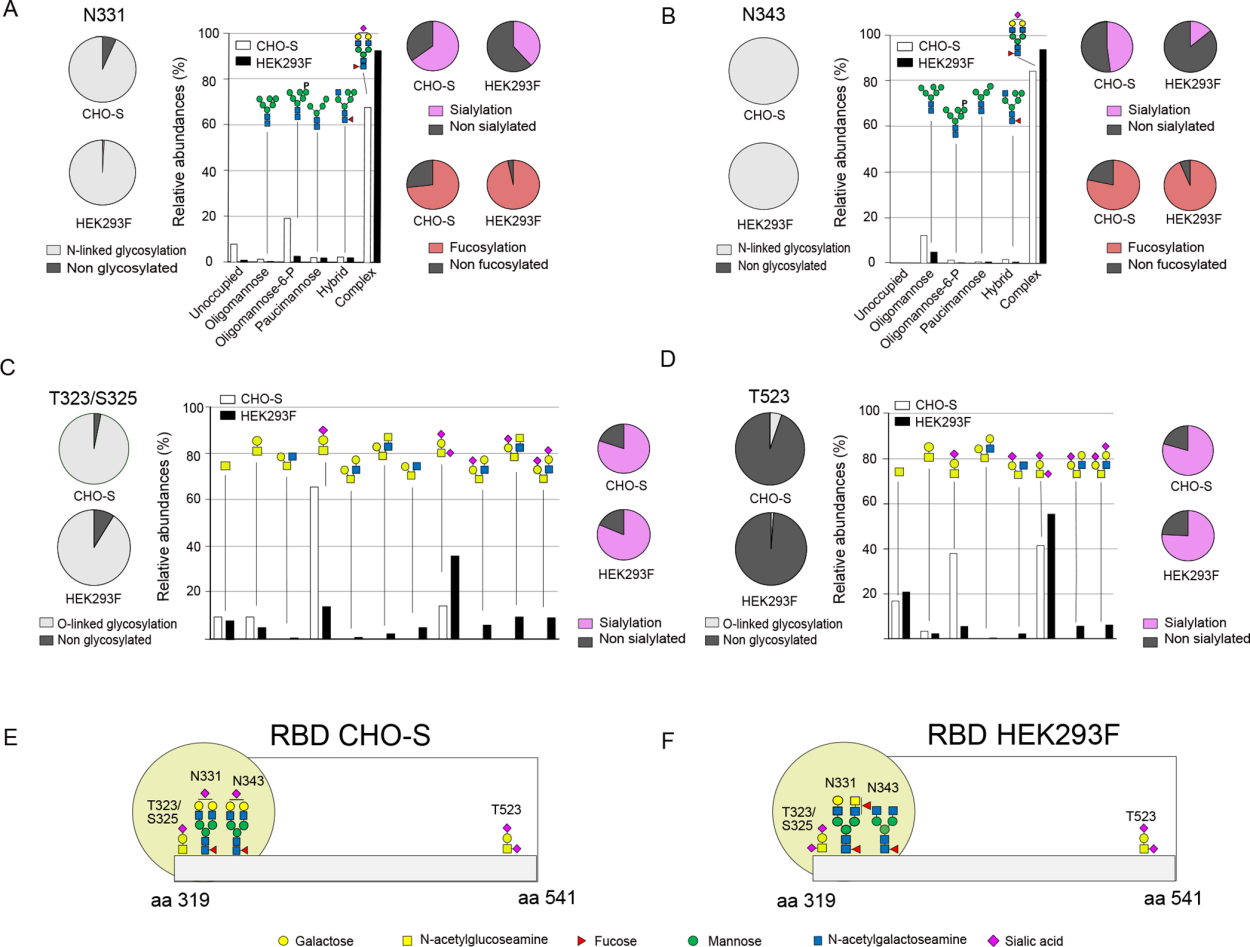
Schematic presentation of glycan distribution at the respective sites of the CHO-S- and HEK293F-produced RBD. The glycan structures are inferred from the obtained mass spectrometry data as well as from previously reported glycan structures and known glycan biosynthesis pathways in mammalian cells. (A) Degree of glycosylation, distribution between the glycan types, degree of sialylation, and degree of fucosylation at site N331 as detected on the CHO-S- and HEK293F-produced RBD. (B) Degree of glycosylation, distribution between the glycan types, degree of sialylation, and degree of fucosylation at site N343 as detected on the CHO-S- and HEK293F-produced RBD. (C) Degree of glycosylation, distribution between glycan compositions, and degree of sialylation at site T323/S325 as detected on the CHO-S- and HEK293F-produced RBD. (D) Degree of glycosylation, distribution between glycan compositions, and degree of sialylation at site T523 as detected on the CHO-S- and HEK293F-produced RBD. (E) Glycosylation of the recombinant RBD produced in CHO-S cells with the most prevalent glycans drawn at the respective site. The yellow circle highlights the glycan hotspot. (F) Glycosylation of the recombinant RBD produced in HEK293F cells with the most prevalent glycans drawn at the respective site. The yellow circle highlights the glycan hotspot.

The recombinant RBD, produced in HEK293F- and CHO-S-cells, respectively, was subjected to nano-LC–MS/MS analysis. We defined the level of occupancies and composition of the N-linked and O-linked glycans present in the RBD. In addition, we suggest N- and O-linked glycan structures based on the observed glycan compositions and the knowledge of the mammalian glycan biosynthesis pathways. The HEK293F-produced RBD showed a nearly complete occupancy for both N-linked sites (99.1 and 100%), while the CHO-S-produced construct presented a partial occupancy of 93.3% for site N331 and a full occupancy for N343 (Figure 1A,B). Complex-type N-linked glycans were the most abundant structure in both cell lines; still, a higher degree of glycans processed to complex type was associated with the HEK293F-cell line, while oligomannose structures were relatively more abundant for the CHO-S-produced protein. The observed CHO-S-produced oligomannose glycans were different at the two sites with N331 displaying higher levels of oligomannose-6-phosphate glycans (Figure 1A,B and Table 1).

**Table 1:** Percentage Distribution of Glycan Types at Sites N331 and N343 When Produced in CHO-S, HEK293F, and Lec3.2.8.1 Cells

	N331			N343		
	CHO-S	HEK293F	Lec3.2.8.1	CHO-S	HEK293F	Lec3.2.8.1
unoccupied	7.8	0.9	2.5	0.0	0.0	0.0
oligomannose	1.3	0.4	76.1	12.1	4.9	79.8
oligomannose-6-P	19.1	2.7	10.5	1.4	0.2	2.3
paucimannose	2.1	2.0	7.4	0.6	0.6	12.4
hybrid	2.5	2.1	2.2	1.6	0.5	5.5
complex	67.3	91.8	1.3	84.3	93.8	0.0

Among the complex-type N-linked glycans, biantennary structures were most frequently found at both positions (Table S1). Despite similar glycan compositions in both cell lines, the fragment spectral evaluation identified prominent differences for glycans produced in CHO-S and HEK293F cells. The major difference was the prominent LacDiNAc-containing structures in the HEK293F-produced RBD, while those were absent in the CHO-S-produced protein (Table S1).

Within a given cell line, the frequency of fucose residues was similar for both sites, while the overall fucosylation was higher for HEK293F compared to CHO-S (Figure 1A,B and Table 2). For HEK293F and CHO-S, fucosylation was observed for paucimannose, hybrid, and complex structures. The Lec3.2.8.1 cell mainly produced the oligomannose structures with fucosylation observed on HexNAc(2)Hex(5). As all glycan groups were observed being fucosylated, all of them were included in calculation of the total fucosylation level. Not only the total fucosylation level but also the degree of fucosylation (number of fucose residues per glycan) differs between the cell lines (Table S2). CHO-S cells predominantly produced monofucosylated structures with the fucose placed at the core, as based on the fragment ion analysis. Multiple fucosylation, with up to four fucose residues per glycan, was observed for the RBD produced in the HEK293F cell line. The attachment of fucose to LacNAc and LacDiNAc was observed based on the fragment spectral evaluation.

**Table 2:** Percentage of Detected Glycans Carrying at Least One Fucose or Sialic Acid at Sites N331 and N343 When Produced in CHO-S, HEK293F, and Lec3.2.8.1 Cells

	N331			N343		
	CHO-S	HEK293F	Lec3.2.8.1	CHO-S	HEK293F	Lec3.2.8.1
fucose^a^	67.5	95.7	31.4	78.4	93.5	6.3
fucose^b^	73.2	96.6	31.4	78.4	93.5	6.3
sialic acid^a^	38.9	35.2	0.0	40.8	13.3	0.0
sialic acid^c^	65.0	38.4	0.0	47.9	14.1	0.0

a The percentage is calculated in relation to all glycosylated forms sharing the same peptide sequence, including nonglycosylated peptides.

b The percentage is calculated in relation of a total amount of all observed glycosylated forms, and unoccupied peptides were excluded when performing the calculations.

c The percentage is calculated in relation of a total amount of all observed hybrid- and complex-type glycoforms, and the unoccupied peptides and all oligomannose glycoforms were excluded from the calculations.

In contrast to fucosylation, the sialylation level, calculated including all glycan groups that can be sialylated, i.e., hybrid- and complex-type glycans, was lower for HEK293F compared to CHO-S (Figure 1A,B and Table 2). Also, the degree of sialylation (number of sialic acid residues per glycan) differed between the cell lines (Table S3). CHO-S cells produced multiple sialylated forms in contrast to HEK293F, where mainly monosialylated structures were observed. The lower sialylation level in glycans produced by HEK293F is likely a result of the extensive fucosylation in this cell type. In both cell lines, the major N-glycan type carrying sialic acid was the complex type and a difference between the sites was noted, with N-linked glycans at position N331 displaying higher sialylation levels.

The O-linked glycans were similar for the two cell types (Figure 1C,D and Table S4). The O-linked glycan close to the NTD of the RBD could not be defined to a single amino acid, due to the absence of fragment ions between the two adjacent potential sites, and thus could be placed either at amino acid position T323 or S325. The site T323/S325 was glycosylated to a high degree (97 and 91%, for CHO-S and HEK293F, respectively), while T523 was scarcely decorated and mainly remained nonglycosylated in both the CHO-S- and HEK293F-produced RBD (5 and 1%, respectively). Comparison of O-linked glycans at the individual sites revealed more extensive processing in the HEK293F-produced protein, while the CHO-S-produced O-linked glycans almost exclusively consisted of core 1 structures (Figure 1C,D). The degree of sialylated structures at site T323/S325 was similar between the cell lines (80 and 82% for CHO-S and HEK293F, respectively), while the degree of monosialylated structures (66 and 35%, respectively) and disialylated structures (15 and 46%, respectively) differed. Similarly, the frequency of sialylated structures at site T523 was similar between CHO-S (80%) and HEK293F (79%) cells. The degree of monosialylation was higher in the CHO-S-produced RBD, as compared to the HEK293F-produced protein (38 and 14%, respectively), while a higher degree of disialylation was seen on the HEK293F-produced RBD (42 and 62%, respectively) (Figure 1C,D).

In summary, the RBD produced in CHO-S cells carried O-linked glycans at two positions although only position T323/S325 appeared to be glycosylated with a high frequency. The main type of O-linked glycan found at this position was a core 1 structure with a single sialic acid at the distal galactose (Figure 1E). This RBD protein also carried two N-linked glycans at positions N331 and N343. The predominant type of N-linked glycan was the biantennary complex type, although many variants of complex-type glycans were found. The RBD produced in HEK293F cells predominantly carried core 1 O-linked glycans with two sialic acids, one attached to the distal galactose and one to the innermost GalNAc residue. The N-linked glycans on the HEK293F RBD were almost exclusively of complex type with a high degree of fucosylation (Figure 1F). To note, all glycan structures presented in Figure 1 are inferred from the obtained mass spectrometry data as well as from previously reported glycan structures and known glycan biosynthesis pathways in mammalian cells. Also, the measurements and calculations of abundances used for values presented in Figure 1, Tables 1 and 2 are described in detail in the Materials and Methods Section, The Analysis of RBD Glycosylation Section, and Tables S7 and S8.

### Evaluation of Convalescent Sera from COVID-19 Patients

**Figure 2:**
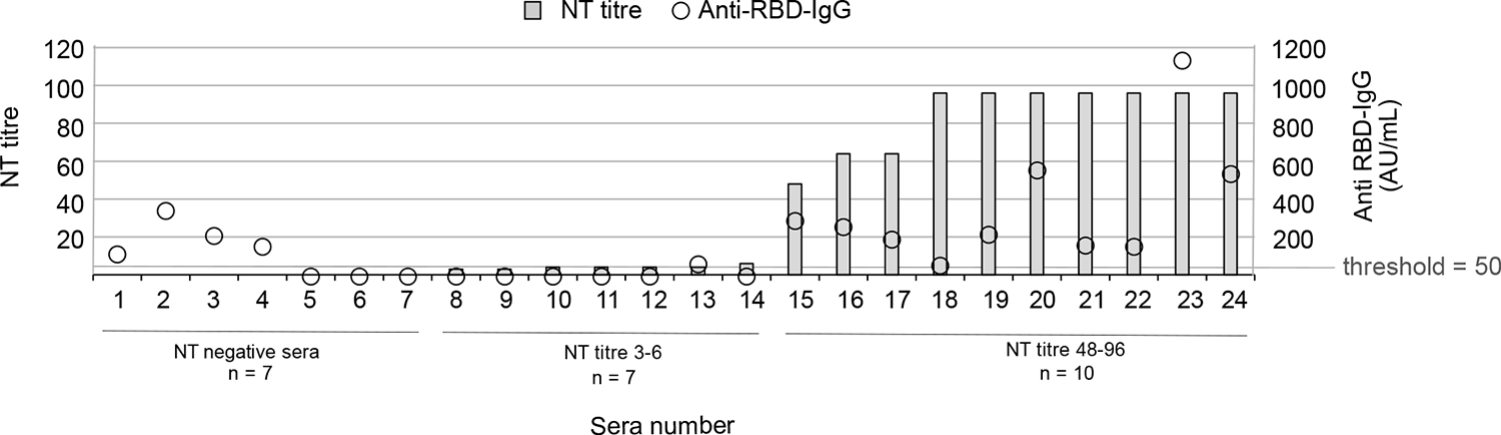
NT-titre (left *y* axis, gray bars) and anti-RBD IgG-levels (right *y* axis, transparent circles) for the 24 characterized serum samples. The sera were divided into three groups based on the neutralizing capability: non-neutralizing (NT-negative, *n* = 7), weakly neutralizing (NT titre 3–6, *n* = 7), and highly neutralizing (NT titre 48–96, *n* = 10). Anti RBD-IgG value ≥50 AU/mL is considered positive.

Serum samples were collected from 24 individuals previously infected with SARS-CoV-2 as determined by a PCR-positive nasopharyngeal sample. Blood samples were collected 25–100 days following positive diagnosis. All sera were characterized with respect to anti-RBD IgG levels and the capability to neutralize a DE-Gbg20 strain of SARS-CoV-2 grown in VERO-cells. Based on the neutralization capability, the sera were divided to three groups: non-neutralizing (NT negative, *n* = 7), weakly neutralizing (NT titre 3–6, *n* = 7), and highly neutralizing (NT titre 48–96, *n* = 10) (Figure 2 and Table S5). High neutralization capability correlated well with high levels of IgG targeting the RBD, as all highly neutralizing sera also were anti-RBD IgG positive, while six of the seven serum samples in the weakly neutralizing group were anti-RBD IgG negative. Interestingly, four out of seven serum samples in the NT negative group were anti-RBD IgG positive.

### Impact of Glycan Structures on Antibody Reactivity against the RBD

To assess the impact of the different types of glycan structures found within the RBD, we removed the N-linked, the O-linked, or a combination of both glycans using enzymatic treatment. Removal of glycans was verified by a size shift on an SDS-page gel, visualized by silver staining (Figure S1A). Based on the glycan structures identified in the present study, the used enzyme combination would in theory result in complete removal of O-linked glycans from the CHO-S- and Lec3.2.8.1-produced RBD and removal of 71.4% of the O-linked glycans from the HEK293F-produced RBD.

**Figure 3:**
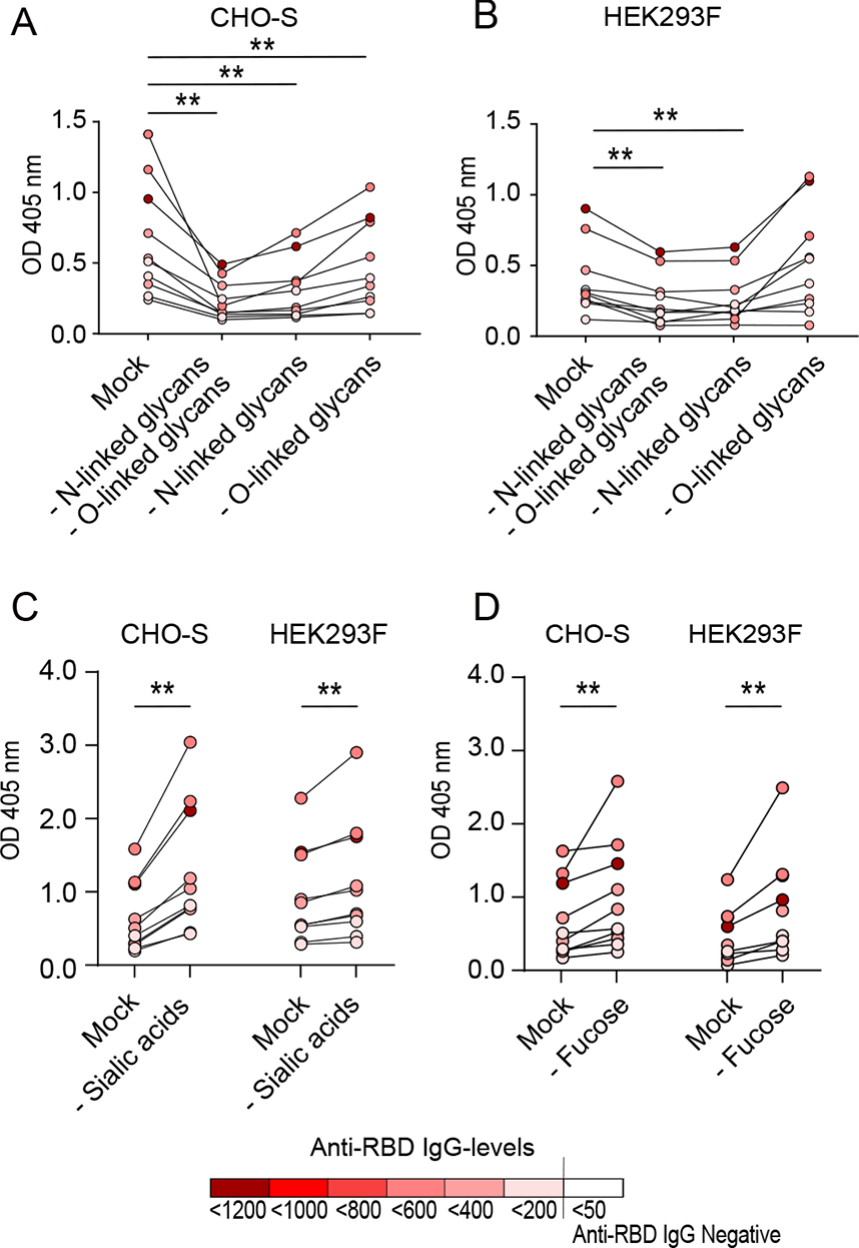
Reactivity of highly neutralizing sera (NT titre 48–96, *n* = 10) against the fully glycosylated RBD (mock treated) and against the deglycosylated RBD produced in CHO-S and HEK293F cells. (A) Removal of both N-linked and O-linked glycans, removal of N-linked glycans alone, or removal of O-linked glycans alone from the RBD produced in CHO-S cells. (B) Removal of both N-linked and O-linked glycans, removal of N-linked glycans alone, or removal of O-linked glycans alone from the RBD produced in HEK293F cells. (C) Removal of sialic acids alone from the RBD produced in CHO-S and HEK293F cells. (D) Removal of fucose alone from the RBD produced in CHO-S and HEK293F cells. Data information: dark red color symbolizes a serum with high levels of anti-RBD IgG, and white color indicates anti-RBD IgG-negative serum (<50 AU/mL). Statistical analysis was performed with the Wilcoxon matched-pair signed rank test, ** = *p* < 0.001.

The effect of glycan removal on the antibody reactivity against the recombinant RBD was tested using the defined serum samples described above. The RBD produced in CHO-S cells elicited a strong reactivity to the highly neutralizing sera, with reduced reactivity following removal of N-linked glycans, O-linked glycans, or a combination of both (Figure 3A). The highly neutralizing sera showed reduced reactivity against the RBD with removed N-linked glycans and the RBD lacking both N- and O-linked glycans produced in HEK293F-cells, while no effect on reactivity against the RBD lacking only O-linked glycans was observed (Figure 3B). The enzymatic treatment utilized to remove O-linked glycans will also remove sialic acid residues from the remaining N-linked glycans. Thus, the observed effects on antibody binding to the RBD lacking O-linked glycans may reflect changes in the N-linked glycans. Weakly neutralizing sera did not show any reactivity to the CHO-S- or the HEK293F-produced RBD regardless of the glycosylation profile (Figure S2A,C). The non-neutralizing serum samples displayed low reactivity against all recombinant RBD with only a minor difference depending on the glycosylation status (Figure S2B,D). The intensity of the reactivity of individual serum samples against the recombinant RBD correlated well with the anti-RBD IgG levels detected in each serum (Figure S3). Only heat treatment for 24 h without addition of enzymes (mock) resulted in significantly higher sera reactivity for the CHO-S-produced RBD compared to the untreated variant, while no difference was noted for the HEK293F- and Lec3.2.8.1-produced RBD (Figure S4).

To further assess the impact of specific glycan residues on antibody reactivity, sialic acids and fucose groups were enzymatically removed from the CHO-S- and HEK293F-produced RBD. SDS-page gel electrophoresis with silver stain and lectin blots were used to confirm the removal of sialic acids or fucose groups. Small, but distinct, size shifts were evident after the enzymatic treatments (Figure S1B,C), and lectin blots using MAL II further verified removal of sialic acids from both the CHO-S- and HEK293F-produced RBD (Figure S5A). Removal of fucose was verified by the use of UEA I-lectin blots (Figure S5B). Removal of sialic acids from the CHO-S-produced RBD resulted in a significant increase in the serum reactivity, as compared to the fully glycosylated RBD. A similar effect was seen following removal of sialic acids from the HEK293F-produced RBD, but the difference was less prominent (Figure 3C). Removal of fucose groups also resulted in a significant increase in serum reactivity for both the CHO-S- and HEK293F-produced RBD, with the HEK293F-produced construct showing a more prominent increase (Figure 3D).

**Figure 4:**
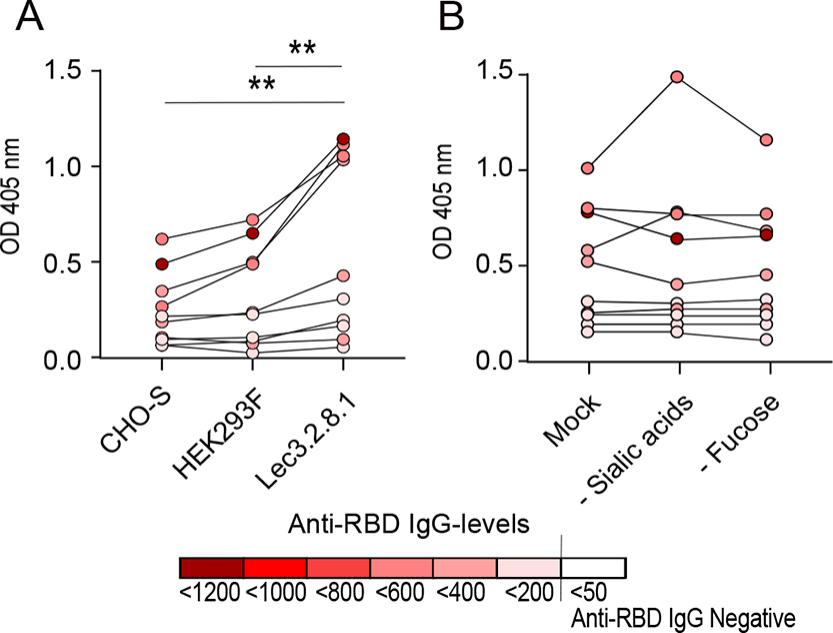
Antibody reactivity of highly neutralizing sera (NT titre 48–96, *n* = 10). (A) Reactivity against the fully glycosylated RBD (untreated) expressed in CHO-S, HEK293F, and Lec3.2.8.1 cells. (B) Reactivity against the fully glycosylated RBD (mock treated) produced in Lec-3.2.8.1 cells, or against the Lec3.2.8.1-produced RBD following enzymatic removal of sialic acids and fucose. Data information: dark red color symbolizes a serum with high levels of anti-RBD IgG, and white color indicates anti-RBD IgG-negative serum (<50 AU/mL). Statisitical analysis was performed with the Wilcoxon matched-pair signed rank test, ** = *p* < 0.001.

To confirm the impact of sialic acids and fucose groups on the antibody reactivity, the RBD was produced in Lec3.2.8.1 cells deficient in synthesis of complex-type glycans. Highly neutralizing serum samples showed a significantly higher reactivity against the RBD produced in Lec3.2.8.1 cells, as compared to the CHO-S- or HEK293F-produced RBD constructs (Figure 4A). As expected, enzymatic removal of sialic acids and fucose from the Lec3.2.8.1-produced RBD did not confer any detectable size shift on an SDS-page gel (Figure S1D) or a change in antibody reactivity by highly neutralizing sera (Figure 4B).

### Glycosylation of the Recombinant RBD Produced in Lec3.2.8.1 Cells

In order to verify that the recombinant RBD produced in Lec3.2.8.1 cells lacked complex-type glycans, it was subjected to nano-LC–MS/MS analysis. Both N-linked sites were found to be glycosylated to a high degree (98 and 88%, respectively). The structural distribution was similar between the sites, with high mannose as the dominating glycan type (Table 1). No sialic acid or end-fucose was found; however, 6% of the structures at site N331 and 31% of the structures at site N343 carried core fucose (Table 2). Position T323/S325 was frequently (98%) decorated with an O-linked glycan, while site T523 was more sparsely decorated (15%). A single HexNAc was the most frequent structure at both O-linked sites (Table S4).

## Discussion

Viruses that infect humans do not carry their own glycosyltransferases but instead rely on the enzymes of the host cell in the processing of their glycans. Hence, viral envelope proteins are therefore glycosylated by the host glycosylation machinery that initially folds and then add glycans to them. This often results in a viral glycosylation profile where glycans protecting antibody epitopes are selected, which does not stimulate a strong immune response. Thus, in order to circumvent the antibody responses, many viruses utilize the host cell glycosylation machinery to cover B-cell epitopes with a dense network of N-linked glycans, which due to physical hindrance shield the epitopes and prevent binding by neutralizing antibodies.^28^ With a few exceptions, N-linked glycans alone rarely act as antibody epitopes,^29^ but the opposite effect has been observed with small O-linked glycans. Olofsson et al. showed that 70% of tested sera against herpes simplex virus (HSV) type 2 contains antibodies targeting a peptide decorated with a single O-linked GalNAc residue. Removal of the glycan moiety diminished this response.^30^ Similarly, our group has previously shown that a single GalNAc-residue added to a naked peptide can alter the antibody binding toward specific domains of the glycoprotein E from the varicella zoster virus (VZV).^31^

It is established that viral glycoproteins are heterogeneously glycosylated when they are expressed either as recombinant proteins in cell culture or after natural infection in cells.^31−33^ This could imply that the antibody response to a viral glycoprotein is more diverse than previously thought. Hence, serum from infected individuals contains a polyclonal antibody pool, which could recognize multiple epitopes, and various glycoforms can constitute parts of these epitopes. The S protein of SARS-CoV-2 is highly glycosylated, with 17 to 22 previously identified sites carrying N-linked glycosylation that can shield B cell epitopes.^15,17,19^ Of these, 2 N-linked glycans are present in the RBD, and Yang et al. identified as many as 10 O-linked glycans in this region, although most of them appeared to be of low abundance and their biological significance is therefore uncertain.^34^ We demonstrate that enzymatic removal of all N-linked and/or O-linked glycans resulted in decreased antibody reactivity of the recombinant SARS-CoV-2 RBD produced in both CHO-S and HEK293F cells. This could indicate (i) that the glycans of the RBD constitute parts of antibody epitopes, (ii) that glycosylation is required for proper protein folding and in maintaining the protein conformation,^35^ or (iii) a combination of both.

The strong immunoreactivity of RBD, as verified by the identification of a multitude of NAbs targeting this domain,^6,8,9^ could be explained by the presence of multiple structural epitopes. If large glycan moieties are lacking, as after enzymatic removal, the folding of the protein could be compromised and thereby also transform structural epitopes, which would render them inaccessible to antibodies present in the serum samples. No linear B-cell epitopes have been identified within the RBD.^36^ However, screening for B cell epitopes is performed with synthetic peptides lacking glycans and would then not be able to identify epitopes that are dependent on the presence of N- or O-linked glycans.

We performed a structural screening of the glycan profile of the recombinant RBD. Overall, our screening is in line with previous studies,^14,16^ but we found significant differences in the amount of sialic acid and fucose content when comparing the RBD produced in CHO-S cells and HEK293F cells, respectively. Interestingly, the CHO-S-produced RBD is also presented with a high degree of mannose-6-phosphate (M-6-P). This structure has previously been observed in the SARS-CoV-2 spike protein when expressed in cell lines but also when isolated from intact viral particles.^33,37^ Mannose-6-phosphate is recognized by the M-6-P receptor present in the trans-Golgi compartment, and it directs tagged proteins to late endosomes/lysosomes. Lysosomal egress dependent on M-6-P has been described for both HSV (26) and VZV (27). Also, SARS-CoV-2 egress mediated by lysosomes has been proposed.^38^ However, we observed only a minor fraction of the peptides carrying M-6-P and to what extent they potentially could contribute to viral particle egress remains to be clarified.

While each glycosite on the recombinant RBD was glycosylated at a similar frequency independent of the production cell line, the RBD produced in HEK293F cells had a higher degree of fucosylation compared to CHO-S cells. Selective removal of the fucose groups resulted in a significantly increased antibody reactivity. While abundant fucosylation was a trait of the HEK293F-produced construct, the RBD produced in CHO-S cells had a larger content of sialic acid moieties. Selective removal of sialic acids enhanced antibody reactivity for both constructs, but the effect was more prominent for the CHO-S produced construct. The use of the Lec3.2.8.1-cell line resulted in RBD with a glycosylation profile completely deficient in sialic acids and end-fucose. The observation of oligomannose structures with core fucosylation in Lec3.2.8.1-cells, although unclear how this type of structure could occur given the known pathways for N-linked glycosylation, has previously been described.^39^ The antibody reactivity toward the Lec3.2.8.1-produced RBD was enhanced, and additional treatment to remove the core fucose did not result in any change in the antibody reactivity. Altogether, these results point to an important function of specific terminal-sugar residues in the antibody reactivity against glycosylated viral antigens and suggest that core fucosylation is of minor importance, despite the report of an NAb that specifically interacts with the core fucose of the N-linked glycan situated on position N343.^9^

In line with our findings that removal of sialic acids leads to increased antibody reactivity, the nonsialylated glycan structures of yeast-cell-produced proteins could possibly be part of the explanation of the highly efficient yeast-produced vaccines against HBV.^40,41^ This suggests that it is possible to optimize recombinantly expressed RBD or S proteins in order to generate effective vaccine candidates. However, important to note is the possibility that immunization with a recombinant expressed subunit vaccine directs the humoral immune response toward B-cell epitopes with species-specific glycosylation profiles. This can possibly result in skewed immunodominance, directing the antibody response toward epitopes that are not exposed after a natural infection with the virus, resulting in disturbed efficiency of the vaccine.^42^ The data presented in this work confirm the necessity of correct glycosylation and show that also small differences in the glycosylation profile of a viral antigen can have a large impact on the reactivity by antibodies generated after a natural infection with SARS-CoV-2. A conscious decision regarding the glycosylation traits of the production cell line could hence affect the antibody response triggered by a recombinant protein. We suggest that the glycosylation characteristics should be considered during the production of recombinant vaccines toward SARS-CoV-2 but also other enveloped viruses, which carry glycoproteins.

## Materials and Methods

### Expression of Recombinant S Protein Constructs

The RBD of the SARS-CoV-2 spike protein (amino acids 319–541) was produced in three cell lines using an expression vector obtained through BEI Resources, NIAID, NIH, which is vector pCAGGS containing SARS-CoV-2, and Wuhan-Hu-1 spike glycoprotein gene RBD with the C-terminal Hexa-Histidine tag (NR-52309) (Table S6).

CHO-S cells (Cat nr R80007, Thermo Fisher Scientific, Waltham, MA) were adapted to grow in suspension in the FectoCHO medium (Polyplus transfection, Illkirch-Graffenstaden, France) at 37 °C in 5% CO_2_ in Optimum Growth flasks (Thomson instrument company, Oceanside, CA) at 130 rpm in a Multitron 4 incubator (Infors, Bottmingen, Schweiz). Lec3.2.8.1 cells (a mutated CHO cell line kindly received from Prof. P Stanley^43^) were cultured under the same conditions. The HEK293 derivate HEK293F cell line (Cat nr R79007, Thermo Fisher Scientific) was cultured in the Freestyle 293 medium. Cells were transfected at 2 × 10^6^ cells/mL using a FectoPro transfection reagent (Polyplus transfection). The temperature was reduced to 32 °C (Lec3.2.8.1) or 31 °C (CHO-S) 4 h post transfection, while transfected HEK293F cells were kept at 37 °C. Protein-containing culture supernatants (800 mL–1 L) were harvested when cell viability was below 80%, which was after 168 h (CHO-S), 74 h (Lec3.2.8.1), or 90 h (HEK293F), filtered using Polydisc AS 0.45 μm (Whatman, Maidstone, UK) and loaded onto a 5 mL HisExcel column (Cytiva, Marlborough, MA). After sample loading, the column was washed with 20 mM sodium phosphate, 0.5 M NaCl, and 30 mM imidazole before elution of the protein using the same buffer with 500 mM imidazole (Lec3.2.8.1-produced RBD) or 300 mM imidazole (CHO-S- and HEK293F-produced RBD). Pooled fractions were concentrated using 10 kDa Vivaspin concentrators (MWCO 10 kDa, Sartorius, Göttingen, Germany), passed over a HiPrep 26/10 desalting column (Cytiva) in phosphate-buffered saline, and finally concentrated again. The Lec3.2.8.1-produced RBD was further purified by gel filtration using a Superose 200 Increase 16/300 GL column (Cytiva) in phosphate-buffered saline. Integrity and purity of the different RBD preparations were checked by SDS-PAGE and Western blot.

### Sample Preparation Prior to Assessment of the Position and Structure of Glycans

The purified RBD preparations from CHO-S, HEK293F, and Lec3.2.8.1 (20 μg each) were diluted with digestion buffer (DB), 1% sodium deoxycholate (SDC) in 50 mM triethylammonium bicarbonate (TEAB) pH 8.0 (Sigma Aldrich, St. Louis, MO), to give protein concentrations of 0.5 μg/μL. The RBD preparations were reduced with 4.5 mM dithiothreitol (DTT) at 56 °C for 30 min and alkylated with 9 mM 2-iodoacetamide in the dark for 30 min at room temperature (RT). The alkylation reactions were then quenched by incubation with DTT (9 mM final concentration) for 15 min at RT. Additional 20 μL of DB was added prior to the proteolytic digest with Pierce MS grade trypsin and Glu-C (overnight at 37 °C, 0.2 and 0.3 μg, respectively). The digested samples were purified using a High Protein and Peptide Recovery Detergent Removal Spin Column (Thermo Fisher Scientific) according to the manufacturer’s instructions. SDC was removed by acidification with 10% trifluoroacetic acid and subsequent centrifugation.

The supernatants were further purified using Pierce peptide desalting spin columns (Thermo Fisher Scientific) according to the manufacturer’s instructions. Each of the purified RBD preparations was divided into three parts: (1) 7.5 μg for nano-LC–MS/MS analysis, (2) 7.5 μg for neuraminidase treatment, and (3) 5 μg for PNGaseF treatment.

For sialic acid removal, RBD preparations were incubated with 1 μL of Sialidase A (GK80040, Agilent, Santa Clara, CA) in 50 μL of provided buffer, overnight at 37 °C. For N-glycan removal, samples were dissolved in 50 μL of 50 mM TEAB and treated with 1 μL of recombinant PNGaseF (Promega, Madison, WI) overnight at 37 °C. All preparations were desalted using Pierce peptide desalting spin columns (Thermo Fisher Scientific) prior to nano-LC–MS/MS analysis.

### Nano-LC–MS/MS Analysis of the Recombinant RBD

The RBD proteolytic preparations were analyzed using a QExactive HF mass spectrometer interfaced with an Easy-nLC1200 liquid chromatography system (Thermo Fisher Scientific). Peptides were trapped using an Acclaim Pepmap 100 C18 trap column (100 μm × 2 cm, particle size 5 μm, Thermo Fischer Scientific) and separated with an in-house packed analytical column (75 μm × 300 mm, particle size 3 μm, Reprosil-Pur C18, Dr. Maisch) using a gradient from 7 to 50% of solvent B over 75 min, followed by an increase to 100% of solvent B for 5 min at a flow of 300 nL/min, where solvent A was 0.2% formic acid (FA) and solvent B was 80% acetonitrile in 0.2% FA. The precursor ion mass spectra were acquired in either 600–2000 *m/z* or 375–1500 *m/z* ranges at a resolution of 120,000. For nano-LC–MS/MS analysis, the instrument operated in data-dependent mode with the 10 most intense ions with charge states 2 to 5 being selected for fragmentation using higher-energy collision dissociation (HCD). The isolation window was set to 3 *m/z* and dynamic exclusion to 20 s. MS/MS spectra were recorded at a resolution of 30,000 with the maximum injection time set to 110 ms. To facilitate glycosylated peptide characterization, multiple injections were acquired with precursor detection in the 600–2000 *m/z* range and different settings for the normalized HCD energies of 22, 28, and 34.

### Glycan Database Search and Data Processing

The acquired data were analyzed using Proteome Discoverer version 2.4 (Thermo Fisher Scientific). Database searches were performed with either Byonic (Protein Metrics, Cupertino, CA) or Sequest as search engines. To evaluate the protein preparation purity, the data were initially searched against the custom database consisting of the Uniprot_Chinese hamster_CHO-K1 cell line database (24,147 proteins), SwissProt_human database (20,342 proteins), and the sequence of expressed RBD protein. For later searches aimed to identify the available glycoforms, the raw data acquired with different HCD energies were searched with Proteome Discoverer/Byonic, with Minora Feature Detector node, against the single RBD protein sequence. Precursor mass tolerance was set to 10 ppm and fragment mass tolerance to 30 ppm. Proteolytic peptides with up to two missed cleavages (combined Trypsin Glu-C cleavage sites) were accepted together with variable modification of methionine oxidation and fixed cysteine alkylation. Several different N-glycan databases were used during the data processing.

The initial N-glycan database contained 227 glycan compositions, where 224 were COVID-19-associated N-glycan compositions reported in GlyConnect Compozitor version: 1.0.0 at SIB Swiss Institute of Bioinformatics | Expasy site plus 3 additional mannose-phosphate containing compositions. This database was used for the analysis of neuraminidase-treated samples. The 45 curated nonsialylated compositions, retrieved from the analysis of neuraminidase-treated preparation, were used to create a new glycan database consisting of 153 glycan compositions for the follow-up analysis of native preparations. An O-glycan database consisted of 6 reported, in GlyConnect Compozitor COVID-19 O-glycan, compositions and was used for the analysis of PNGaseF-treated samples.

All glycopeptide identifications were manually evaluated prior to the final assignment of the observed glycosylation forms. The data, acquired with the normalized HCD energy of 22, were used for oxonium ion evaluation to suggest glycan structures for the observed compositions. The extracted ion chromatogram (EIC) peak intensities of the observed glycoforms were used to calculate their relative abundances. For the N-glycopeptide analysis, the relative abundances were calculated using average EIC values from three injections and are expressed as percent of the total signal for all modified and nonmodified forms. For the O-glycopeptide analysis, two injections were acquired for each PNGAseF-treated sample with precursor ions measured at 375–1500 and 600–2000 *m/z*. The acquisition in the 375–1500 *m/z* range provided the best detection of O-glycosylated peptides and was used for further data evaluation The O-glycopeptide intensities were calculated based on single injection, and no CV (%) calculations are available for their measurements.

### Analysis of RBD Glycosylation

The RBD domain contains two canonical NXS/TN-glycosylation sites, N331 and N343. Proteolytic digest using a combination of Trypsin and Glu-C was selected to access these sites. No glycopeptide enrichment was performed prior to LC–MS/MS, to avoid selective enrichment of specific glycoforms. Instead, to facilitate detection of N-glycopeptides, precursor ions were acquired in the 600–2000 *m/z* range. The acquired LC–MS/MS data were first evaluated for the presence of oxonium ions to confirm that glycopeptides were observed for each site and to evaluate their potential glycan compositions. Both sites were found to be glycosylated in all three expression systems. The expected HexNAc and HexNAcHex oxonium ions, *m/z* 204 and 365, were found for the RBD produced in all three cell lines. The NeuAc oxonium ions (*m/z* 274 and 292) were observed for the RBD expressed in CHO-S and HEK293F but not in Lec3.2.8.1 cell lines. The presence of oligomanose-6-P structures was suggested by the presence of oxonium ions at *m/z* 243, observed for all three RBD preparations.

The initial data evaluation revealed high heterogeneity at both N-glycosylation sites with possibilities of both multifucosylated and multisialylated structures. Therefore, native RBD preparations from CHO-S and HEK293F preparations were further treated with neuraminidase, prior to the first round of the site-specific glycosylation analysis. The removal of sialic acid served several purposes. (i) It decreased site heterogeneity and thus improved the detection of the existing glycoforms. (ii) It simplified identification of multifucosylated structures. (iii) In addition, it facilitated relative quantification of the site microheterogeneity. In the positive ion mode, the ionization of glycopeptide occurs at the polypeptide chain, thus allowing one to use the observed signal intensities for relative glycoform quantification at the same polypeptide base.^44^ The use of glycopeptide signal intensities for both neutral and sialylated glycoforms detected by LC-MS/MS has been shown to have good correlation with quantification carried on AB-labeled glycans.^45^ We commonly observed different charge state distributions as well as later retention times for sialylated glycoforms compared to neutral glycoforms, and therefore, we always perform glycan profile evaluation on both native and neuraminidase-treated preparations.

To minimize the effect of ionization efficiencies, we first used LC-MS/MS data acquired for the neuraminidase-treated samples to compare site-specific glycan profiles of the different RBD preparations. This included the evaluation of glycan-type distribution at each site, the antenna distribution within complex glycans, and the degree of fucosylation. Each RBD preparation was analyzed at least three times with identical MS1 settings but different fragmentation energies in MS2 to facilitate glycoform identifications. The EIC peak intensities were used to determine the glycoform abundances. The average values from three injections were used to calculate glycoform abundances expressed as percent of total signal for all modified and nonmodified peptides sharing the same amino acid sequence (Table S7). These data were used to calculate the values for glycan-type distribution at each site, the antenna distribution within complex glycans, and the degree of fucosylation presented in Figure 1, Tables 1, 2, and S3. The MS2 data were acquired at different collision energies and were used to evaluate the fucose position and antenna compositions including the suggested presence of LacDiNac. The native RBD preparations were then used to evaluate the degree of sialylation for all hybrid and complex structures observed in neuraminidase-treated preparations. The glycoform abundances were calculated as average of three injections (Table S8) and were used to calculate sialylation levels for each site presented in Tables 2 and S4.

To evaluate the presence of O-glycans, the RBD preparations were treated with PNGaseF. For each sample, two injections were acquired with precursor ions measured at 375–1500 and 600–2000 *m/z*. The acquisition in the 375–1500 *m/z* range provided the best detection of O-glycosylated peptides and was used for further data evaluation and calculations presented in Figure 1 and Table S4. The presented values were calculated based on a single injection, and no CV (%) values are available for their measurements.

### Deglycosylation of the Recombinant RBD Using Glycosidase Treatment

Removal of N-linked glycans was performed using PNGaseF (New England Biolabs, Ipswich, USA) at a concentration of 125 U/μg protein. Removal of O-linked glycans was performed using O-glycosidase (New England Biolabs, 20,000 U/μg protein), α2-3,6,8 neuraminidase (New England Biolabs, 25 U/μg protein), and α-*N*-acetyl-galactosaminidase (New England Biolabs, 10 U/μg protein). Removal of both N-linked and O-linked glycans was performed using PNGaseF (New England Biolabs, 125 U/μg protein), O-glycosidase (New England Biolabs, 20,000 U/μg protein), α2-3,6,8 neuraminidase (New England Biolabs, 25 U/μg protein), and α-*N*-acetyl-galactosaminidase (New England Biolabs, 10 U/μg protein). Removal of sialic acids was performed using the α2-3,6,8 neuraminidase (New England Biolabs, 50 U/μg protein). Removal of fucose was performed using α1-2,4,5,6 fucosidase O (New England Biolabs, 2 U/μg protein) and α1-3,4 fucosidase (New England Biolabs, 4 U/μg protein). All enzymatic reactions were performed as a 1-step reaction with 1× Glycobuffer 2 (New England Biolabs) and 10 μg of RBD produced in CHO-S-, HEK293F-, or Lec3.2.8.1 cells and incubation at 37 °C for 24 h. As heat-treated controls, peptides were incubated at 37 °C for 24 h but without additional enzymes.

### Gel Electrophoresis and In-Gel Staining of the Glycosidase-Treated Recombinant RBD

To control efficiency of the enzymatic treatment, 5 μg of the enzyme-treated products or controls were run on a NuPage 4–12% Bis-Tris gel (Invitrogen, Carlsbad, USA) at 100 V for 60 min using an EI9001-XCELL II Mini Cell (Novex, San Diego, CA) together with a Powerease 500 (Novex) and subsequently stained with a SilverQuest Stain kit (Invitrogen) according to the instructions from the manufacturer.

### Lectin Blot of the Glycosidase-Treated Recombinant RBD

First, 2 μg of RBD produced in CHO-S or HEK293F cells was treated with neuraminidase, fucosidase, or mock-treated and separated in NuPAGE Bis-Tris 4–12% gels (Invitrogen) using MOPS buffer (Invitrogen) at 100 V. Separated proteins were blotted to the polyvinylidene difluoride membrane (Immobilon-FL 0.45 μm, Merck Millipore, Burlington, MA) using SemiDry Transblot SD (Bio-Rad Laboratories, Hercules, CA). Membranes were blocked with 2% BSA Factor V (Sigma-Aldrich) and 0.1% Tween-20 (VWR chemicals, Radnor, PA) in phosphate-buffered saline (PBS, Medicago, Uppsala, Sweden) (PBS-BSA-T) and then incubated with biotinylated lectin, either 5 μg/mL MALII or 3 μg/mL UEA (both from Vector laboratories, Burlingame, CA), in PBS-BSA-T at 4 °C for 16–20 h. Membranes were then washed three times with PBS + 0.1% Tween-20, incubated with Streptavidin-alkaline phosphatase diluted 1/2000 (Southern Biotech) for 1 h at 20–22 °C, and washed again. Membranes were developed using BCIP/NBT (Sigma-Aldrich) for 1 min.

### Levels of Human Anti-SARS-CoV-2 IgG Antibodies in Convalescent Serum Samples

Serum samples from SARS-CoV-2 convalescent individuals (*n* = 24) were obtained from the department of Clinical Microbiology, Sahlgrenska University Hospital, Gothenburg, Sweden. Samples were collected between 06-03-2020 and 08-27-2020, 25–100 days following a positive PCR-test. Serum was stored at −80 °C until use. The SARS-CoV-2 IgG II Quant assay is a chemiluminescent microparticle immunoassay used for quantitative determination of IgG antibodies to SARS-CoV-2 in human serum and plasma on an ARCHITECT System (Abbott Laboratories, Chicago, IL). The assay measures IgG binding to the RBD of the S-protein. IgG concentrations ≥50 antibody units (AU)/mL were defined as positive.

### Viral CPE Neutralization Assay

The titre of neutralizing antibodies against SARS-CoV-2 in the patient sera was determined against the DE-Gbg20 viral strain (NCBI GenBank ID: MW092768) at a titre of 10^–6^. A 50% tissue culture infectious dose (TCID50) assay was performed as defined by Reed and Muench.^46^ All sera were heat inactivated at 56 °C for 30 min before twofold serial dilution in serum-free Dulbecco’s Modified Eagle Medium (DMEM) with 100TCID50 DEGbg20 followed by incubation for 2 h at 37 °C. The virus–antibody mixture was added to a monolayer of VERO CCL-81 cells grown in 96 well-plates in DMEM supplemented with 2% penicillin–streptomycin and 2% fetal calf serum. The plates were incubated for 72 h at 37 °C with 5% CO_2_. The neutralizing titre for each serum was defined at the highest serum dilution at which 50% of the added virus was neutralized.

### Anti-SARS-CoV-2 Antibody Reactivity Assay

The antibody reactivities toward glycosidase-treated proteins were assessed using an enzyme-linked immunosorbent assay (ELISA). Briefly, Nunc Maxisorp 96-well plates (Thermo Fischer Scientific) were coated with 0.1 μg of glycosidase-treated peptides or heat-treated controls diluted in carbonate buffer (pH 9.6). Coating was performed overnight at 4 °C followed by washing three times with 0.05% tween20 in PBS. The plates were blocked in 2% milk for 30 min at RT prior to addition of sera (diluted 1:100 in 1% milk in PBS with 0.05% tween20) and 1.5 h incubation at 37 °C. The plates were washed three times before addition of alkaline phosphatase-conjugated goat antihuman IgG (Jackson ImmunoResearch, Cambridgeshire, UK) diluted 1:1000 in 1% milk in PBS with 0.05% tween. After 1.5 h incubation at 37 °C, the plate was washed six times and 1 mg/mL *p*-nitrophenylphosphate (Medicago, Danmarks-Berga, Sweden) in diethanolamine substrate buffer was added. The plates were incubated in the dark for 30 min before spectrophotometric measurement at 405 nm.

### Statistics

For the comparison of antibody reactivity as determined by ELISA, the Wilcoxon matched-pair signed rank test was used. The comparison between anti-RBD IgG levels and antibody reactivity toward recombinant RBD was done with Pearson correlation coefficients, assuming normal distribution. All statistical analyses were performed using the Graphpad Prism software version 9.3.1 (GraphPad Software Inc., San Diego, CA, USA).

### Ethical Statement

The study was approved by the ethical review board in Gothenburg (Dnr: 2021-02252).

## Supporting Information Available

Figures S1−S5 and Tables S1−S6 (PDF)
Table S7 (XLSX)
Table S8 (XLSX)

## References

[ref1] Rydyznski Moderbacher C.; Ramirez S. I.; Dan J. M.; Grifoni A.; Hastie K. M.; Weiskopf D.; Belanger S.; Abbott R. K.; Kim C.; Choi J.; Kato Y.; Crotty E. G.; Kim C.; Rawlings S. A.; Mateus J.; Tse L. P. V.; Frazier A.; Baric R.; Peters B.; Greenbaum J.; Ollmann Saphire E.; Smith D. M.; Sette A.; Crotty S. Antigen-Specific Adaptive Immunity to SARS-CoV-2 in Acute COVID-19 and Associations with Age and Disease Severity. Cell 2020, 183, 996–1012.e19. 10.1016/j.cell.2020.09.038. 33010815 PMC7494270

[ref2] Zhao J.; Yuan Q.; Wang H.; Liu W.; Liao X.; Su Y.; Wang X.; Yuan J.; Li T.; Li J.; Qian S.; Hong C.; Wang F.; Liu Y.; Wang Z.; He Q.; Li Z.; He B.; Zhang T.; Fu Y.; Ge S.; Liu L.; Zhang J.; Xia N.; Zhang Z. Antibody Responses to SARS-CoV-2 in Patients With Novel Coronavirus Disease 2019. Clin. Infect. Dis. 2020, 71, 2027–2034. 10.1093/cid/ciaa344. 32221519 PMC7184337

[ref3] Lou B.; Li T. D.; Zheng S. F.; Su Y. Y.; Li Z. Y.; Liu W.; Yu F.; Ge S. X.; Zou Q. D.; Yuan Q.; Lin S.; Hong C. M.; Yao X. Y.; Zhang X. J.; Wu D. H.; Zhou G. L.; Hou W. H.; Li T. T.; Zhang Y. L.; Zhang S. Y.; Fan J.; Zhang J.; Xia N. S.; Chen Y. Serology characteristics of SARS-CoV-2 infection after exposure and post-symptom onset. Eur. Respir. J. 2020, 56, 2000763 10.1183/13993003.00763-2020. 32430429 PMC7401320

[ref4] Kellam P.; Barclay W. The dynamics of humoral immune responses following SARS-CoV-2 infection and the potential for reinfection. J. Gen. Virol. 2020, 101, 791–797. 10.1099/jgv.0.001439. 32430094 PMC7641391

[ref5] Plotkin S. A.; Plotkin S. A. Correlates of Vaccine-Induced Immunity. Clin. Infect. Dis. 2008, 47, 401–409. 10.1086/589862. 18558875

[ref6] Tortorici M. A.; Beltramello M.; Lempp F. A.; Pinto D.; Dang H. V.; Rosen L. E.; McCallum M.; Bowen J.; Minola A.; Jaconi S.; Zatta F.; De Marco A.; Guarino B.; Bianchi S.; Lauron E. J.; Tucker H.; Zhou J.; Peter A.; Havenar-Daughton C.; Wojcechowskyj J. A.; Case J. B.; Chen R. E.; Kaiser H.; Montiel-Ruiz M.; Meury M.; Czudnochowski N.; Spreafico R.; Dillen J.; Ng C.; Sprugasci N.; Culap K.; Benigni F.; Abdelnabi R.; Foo S.-Y. C.; Schmid M. A.; Cameroni E.; Riva A.; Gabrieli A.; Galli M.; Pizzuto M. S.; Neyts J.; Diamond M. S.; Virgin H. W.; Snell G.; Corti D.; Fink K.; Veesler D. Ultrapotent human antibodies protect against SARS-CoV-2 challenge via multiple mechanisms. Science 2020, 370, 950–957. 10.1126/science.abe3354. 32972994 PMC7857395

[ref7] Ju B.; Zhang Q.; Ge J.; Wang R.; Sun J.; Ge X.; Yu J.; Shan S.; Zhou B.; Song S.; Tang X.; Yu J.; Lan J.; Yuan J.; Wang H.; Zhao J.; Zhang S.; Wang Y.; Shi X.; Liu L.; Zhao J.; Wang X.; Zhang Z.; Zhang L. Human neutralizing antibodies elicited by SARS-CoV-2 infection. Nature 2020, 584, 115–119. 10.1038/s41586-020-2380-z. 32454513

[ref8] Wang C.; Li W.; Drabek D.; Okba N. M. A.; van Haperen R.; Osterhaus A.; van Kuppeveld F. J. M.; Haagmans B. L.; Grosveld F.; Bosch B. J. A human monoclonal antibody blocking SARS-CoV-2 infection. Nat. Commun. 2020, 11, 2251 10.1038/s41467-020-16256-y. 32366817 PMC7198537

[ref9] Pinto D.; Park Y. J.; Beltramello M.; Walls A. C.; Tortorici M. A.; Bianchi S.; Jaconi S.; Culap K.; Zatta F.; De Marco A.; Peter A.; Guarino B.; Spreafico R.; Cameroni E.; Case J. B.; Chen R. E.; Havenar-Daughton C.; Snell G.; Telenti A.; Virgin H. W.; Lanzavecchia A.; Diamond M. S.; Fink K.; Veesler D.; Corti D. Cross-neutralization of SARS-CoV-2 by a human monoclonal SARS-CoV antibody. Nature 2020, 583, 290–295. 10.1038/s41586-020-2349-y. 32422645

[ref10] Chi X.; Yan R.; Zhang J.; Zhang G.; Zhang Y.; Hao M.; Zhang Z.; Fan P.; Dong Y.; Yang Y.; Chen Z.; Guo Y.; Zhang J.; Li Y.; Song X.; Chen Y.; Xia L.; Fu L.; Hou L.; Xu J.; Yu C.; Li J.; Zhou Q.; Chen W. A neutralizing human antibody binds to the N-terminal domain of the Spike protein of SARS-CoV-2. Science 2020, 369, 650–655. 10.1126/science.abc6952. 32571838 PMC7319273

[ref11] Li W.; Moore M. J.; Vasilieva N.; Sui J.; Wong S. K.; Berne M. A.; Somasundaran M.; Sullivan J. L.; Luzuriaga K.; Greenough T. C.; Choe H.; Farzan M. Angiotensin-converting enzyme 2 is a functional receptor for the SARS coronavirus. Nature 2003, 426, 450–454. 10.1038/nature02145. 14647384 PMC7095016

[ref12] Delmas B.; Laude H. Assembly of coronavirus spike protein into trimers and its role in epitope expression. J. Virol. 1990, 64, 5367–5375. 10.1128/jvi.64.11.5367-5375.1990. 2170676 PMC248586

[ref13] Cavanagh D. Coronavirus IBV: structural characterization of the spike protein. J. Gen. Virol. 1983, 64, 2577–2583. 10.1099/0022-1317-64-12-2577. 6319549

[ref14] Shajahan A.; Supekar N. T.; Gleinich A. S.; Azadi P. Deducing the N- and O-glycosylation profile of the spike protein of novel coronavirus SARS-CoV-2. Glycobiology 2020, 30, 981 10.1093/glycob/cwaa042. 32363391 PMC7239183

[ref15] Allen J. D.; Chawla H.; Samsudin F.; Zuzic L.; Shivgan A. T.; Watanabe Y.; He W.-T.; Callaghan S.; Song G.; Yong P.; Brouwer P. J. M.; Song Y.; Cai Y.; Duyvesteyn H. M. E.; Malinauskas T.; Kint J.; Pino P.; Wurm M. J.; Frank M.; Chen B.; Stuart D. I.; Sanders R. W.; Andrabi R.; Burton D. R.; Li S.; Bond P. J.; Crispin M. Site-Specific Steric Control of SARS-CoV-2 Spike Glycosylation. Biochemistry 2021, 60, 2153–2169. 10.1021/acs.biochem.1c00279. 34213308 PMC8262170

[ref16] Watanabe Y.; Allen J. D.; Wrapp D.; McLellan J. S.; Crispin M. Site-specific glycan analysis of the SARS-CoV-2 spike. Science 2020, 369, 330–333. 10.1126/science.abb9983. 32366695 PMC7199903

[ref17] Sanda M.; Morrison L.; Goldman R. N- and O-Glycosylation of the SARS-CoV-2 Spike Protein. Anal. Chem. 2021, 93, 2003–2009. 10.1021/acs.analchem.0c03173. 33406838 PMC7805595

[ref18] Bagdonaite I.; Thompson A. J.; Wang X.; Søgaard M.; Fougeroux C.; Frank M.; Diedrich J. K.; Yates J. R.; Salanti A.; Vakhrushev S. Y.; Paulson J. C.; Wandall H. H. Site-specific O-glycosylation analysis of SARS-CoV-2 spike protein produced in insect and human cells. Viruses 2021, 13, 551 10.3390/v13040551. 33806155 PMC8064498

[ref19] Antonopoulos A.; Broome S.; Sharov V.; Ziegenfuss C.; Easton R. L.; Panico M.; Dell A.; Morris H. R.; Haslam S. M. Site-specific characterization of SARS-CoV-2 spike glycoprotein receptor-binding domain. Glycobiology 2021, 31, 181–187. 10.1093/glycob/cwaa085. 32886791 PMC7499654

[ref20] Croset A.; Delafosse L.; Gaudry J.-P.; Arod C.; Glez L.; Losberger C.; Begue D.; Krstanovic A.; Robert F.; Vilbois F.; Chevalet L.; Antonsson B. Differences in the glycosylation of recombinant proteins expressed in HEK and CHO cells. J. Biotechnol. 2012, 161, 336–348. 10.1016/j.jbiotec.2012.06.038. 22814405

[ref21] Chawla H.; Jossi S. E.; Faustini S. E.; Samsudin F.; Allen J. D.; Watanabe Y.; Newby M. L.; Marcial-Juárez E.; Lamerton R. E.; McLellan J. S.; Bond P. J.; Richter A. G.; Cunningham A. F.; Crispin M. Glycosylation and Serological Reactivity of an Expression-enhanced SARS-CoV-2 Viral Spike Mimetic. J. Mol. Biol. 2022, 434, 167332 10.1016/j.jmb.2021.167332. 34717971 PMC8550889

[ref22] Huang H. Y.; Liao H. Y.; Chen X.; Wang S. W.; Cheng C. W.; Shahed-Al-Mahmud M.; Liu Y. M.; Mohapatra A.; Chen T. H.; Lo J. M.; Wu Y. M.; Ma H. H.; Chang Y. H.; Tsai H. Y.; Chou Y. C.; Hsueh Y. P.; Tsai C. Y.; Huang P. Y.; Chang S. Y.; Chao T. L.; Kao H. C.; Tsai Y. M.; Chen Y. H.; Wu C. Y.; Jan J. T.; Cheng T. R.; Lin K. I.; Ma C.; Wong C. H. Vaccination with SARS-CoV-2 spike protein lacking glycan shields elicits enhanced protective responses in animal models. Sci. Transl. Med. 2022, 14, eabm0899 10.1126/scitranslmed.abm0899. 35230146 PMC9802656

[ref23] Casalino L.; Gaieb Z.; Goldsmith J. A.; Hjorth C. K.; Dommer A. C.; Harbison A. M.; Fogarty C. A.; Barros E. P.; Taylor B. C.; McLellan J. S.; Fadda E.; Amaro R. E. Beyond Shielding: The Roles of Glycans in the SARS-CoV-2 Spike Protein. ACS Cent. Sci. 2020, 6, 1722–1734. 10.1021/acscentsci.0c01056. 33140034 PMC7523240

[ref24] Sztain T.; Ahn S. H.; Bogetti A. T.; Casalino L.; Goldsmith J. A.; Seitz E.; McCool R. S.; Kearns F. L.; Acosta-Reyes F.; Maji S.; Mashayekhi G.; McCammon J. A.; Ourmazd A.; Frank J.; McLellan J. S.; Chong L. T.; Amaro R. E. A glycan gate controls opening of the SARS-CoV-2 spike protein. Nat. Chem. 2021, 13, 963–968. 10.1038/s41557-021-00758-3. 34413500 PMC8488004

[ref25] Chawla H.; Fadda E.; Crispin M. Principles of SARS-CoV-2 glycosylation. Curr. Opin. Struct. Biol. 2022, 75, 102402 10.1016/j.sbi.2022.102402. 35717706 PMC9117168

[ref26] Thai R.; Moine G.; Desmadril M.; Servent D.; Tarride J. L.; Ménez A.; Léonetti M. Antigen stability controls antigen presentation. J. Biol. Chem. 2004, 279, 50257–50266. 10.1074/jbc.M405738200. 15364925

[ref27] Tokatlian T.; Read B. J.; Jones C. A.; Kulp D. W.; Menis S.; Chang J. Y. H.; Steichen J. M.; Kumari S.; Allen J. D.; Dane E. L.; Liguori A.; Sangesland M.; Lingwood D.; Crispin M.; Schief W. R.; Irvine D. J. Innate immune recognition of glycans targets HIV nanoparticle immunogens to germinal centers. Science 2019, 363, 649–654. 10.1126/science.aat9120. 30573546 PMC6420719

[ref28] Grant O. C.; Montgomery D.; Ito K.; Woods R. J. Analysis of the SARS-CoV-2 spike protein glycan shield reveals implications for immune recognition. Sci. Rep. 2020, 10, 14991 10.1038/s41598-020-71748-7. 32929138 PMC7490396

[ref29] Raska M.; Takahashi K.; Czernekova L.; Zachova K.; Hall S.; Moldoveanu Z.; Elliott M. C.; Wilson L.; Brown R.; Jancova D.; Barnes S.; Vrbkova J.; Tomana M.; Smith P. D.; Mestecky J.; Renfrow M. B.; Novak J. Glycosylation patterns of HIV-1 gp120 depend on the type of expressing cells and affect antibody recognition. J. Biol. Chem. 2010, 285, 20860–20869. 10.1074/jbc.M109.085472. 20439465 PMC2898351

[ref30] Olofsson S.; Blixt O.; Bergstrom T.; Frank M.; Wandall H. H., Viral O-GalNAc peptide epitopes: a novel potential target in viral envelope glycoproteins. In Reviews in medical virology, ed.; 2016; 26, 34–48. 26524377 10.1002/rmv.1859

[ref31] Nordén R.; Nilsson J.; Samuelsson E.; Risinger C.; Sihlbom C.; Blixt O.; Larson G.; Olofsson S.; Bergstrom T. Recombinant Glycoprotein E of Varicella Zoster Virus Contains Glycan-Peptide Motifs That Modulate B Cell Epitopes into Discrete Immunological Signatures. Int. J. Mol. Sci. 2019, 20, 954 10.3390/ijms20040954. PMC6412795 30813247

[ref32] Nordén R.; Halim A.; Nyström K.; Bennett E. P.; Mandel U.; Olofsson S.; Nilsson J.; Larson G. O-linked glycosylation of the mucin domain of the herpes simplex virus type 1-specific glycoprotein gC-1 is temporally regulated in a seed-and-spread manner. J. Biol. Chem. 2015, 290, 5078–5091. 10.1074/jbc.M114.616409. 25548287 PMC4335243

[ref33] Brun J.; Vasiljevic S.; Gangadharan B.; Hensen M.; Chandran A. V.; Hill M. L.; Kiappes J. L.; Dwek R. A.; Alonzi D. S.; Struwe W. B.; Zitzmann N. Assessing Antigen Structural Integrity through Glycosylation Analysis of the SARS-CoV-2 Viral Spike. ACS Cent. Sci. 2021, 7, 586–593. 10.1021/acscentsci.1c00058. 34056088 PMC8029450

[ref34] Yang J.; Wang W.; Chen Z.; Lu S.; Yang F.; Bi Z.; Bao L.; Mo F.; Li X.; Huang Y.; Hong W.; Yang Y.; Zhao Y.; Ye F.; Lin S.; Deng W.; Chen H.; Lei H.; Zhang Z.; Luo M.; Gao H.; Zheng Y.; Gong Y.; Jiang X.; Xu Y.; Lv Q.; Li D.; Wang M.; Li F.; Wang S.; Wang G.; Yu P.; Qu Y.; Yang L.; Deng H.; Tong A.; Li J.; Wang Z.; Yang J.; Shen G.; Zhao Z.; Li Y.; Luo J.; Liu H.; Yu W.; Yang M.; Xu J.; Wang J.; Li H.; Wang H.; Kuang D.; Lin P.; Hu Z.; Guo W.; Cheng W.; He Y.; Song X.; Chen C.; Xue Z.; Yao S.; Chen L.; Ma X.; Chen S.; Gou M.; Huang W.; Wang Y.; Fan C.; Tian Z.; Shi M.; Wang F.-S.; Dai L.; Wu M.; Li G.; Wang G.; Peng Y.; Qian Z.; Huang C.; Lau J. Y.-N.; Yang Z.; Wei Y.; Cen X.; Peng X.; Qin C.; Zhang K.; Lu G.; Wei X. A vaccine targeting the RBD of the S protein of SARS-CoV-2 induces protective immunity. Nature 2020, 586, 572–577. 10.1038/s41586-020-2599-8. 32726802

[ref35] Shental-Bechor D.; Levy Y. Effect of glycosylation on protein folding: a close look at thermodynamic stabilization. Proc. Natl. Acad. Sci. U. S. A. 2008, 105, 8256–8261. 10.1073/pnas.0801340105. 18550810 PMC2448824

[ref36] Li Y.; Ma M.-L.; Lei Q.; Wang F.; Hong W.; Lai D.-Y.; Hou H.; Xu Z.-W.; Zhang B.; Chen H.; Yu C.; Xue J.-B.; Zheng Y.-X.; Wang X.-N.; Jiang H.-W.; Zhang H.-N.; Qi H.; Guo S.-J.; Zhang Y.; Lin X.; Yao Z.; Wu J.; Sheng H.; Zhang Y.; Wei H.; Sun Z.; Fan X.; Tao S.-C. Linear epitope landscape of the SARS-CoV-2 Spike protein constructed from 1,051 COVID-19 patients. Cell Rep. 2021, 34, 108915 10.1016/j.celrep.2021.108915. 33761319 PMC7953450

[ref37] Gstöttner C.; Zhang T.; Resemann A.; Ruben S.; Pengelley S.; Suckau D.; Welsink T.; Wuhrer M.; Domínguez-Vega E. Structural and Functional Characterization of SARS-CoV-2 RBD Domains Produced in Mammalian Cells. Anal. Chem. 2021, 93, 6839–6847. 10.1021/acs.analchem.1c00893. 33871970 PMC8078197

[ref38] Ghosh S.; Dellibovi-Ragheb T. A.; Kerviel A.; Pak E.; Qiu Q.; Fisher M.; Takvorian P. M.; Bleck C.; Hsu V. W.; Fehr A. R.; Perlman S.; Achar S. R.; Straus M. R.; Whittaker G. R.; de Haan C. A. M.; Kehrl J.; Altan-Bonnet G.; Altan-Bonnet N. β-Coronaviruses Use Lysosomes for Egress Instead of the Biosynthetic Secretory Pathway. Cell 2020, 183, 1520–1535.e14. 10.1016/j.cell.2020.10.039. 33157038 PMC7590812

[ref39] North S. J.; Huang H. H.; Sundaram S.; Jang-Lee J.; Etienne A. T.; Trollope A.; Chalabi S.; Dell A.; Stanley P.; Haslam S. M. Glycomics profiling of Chinese hamster ovary cell glycosylation mutants reveals N-glycans of a novel size and complexity. J. Biol. Chem. 2010, 285, 5759–5775. 10.1074/jbc.M109.068353. 19951948 PMC2820803

[ref40] Ho J. K.-T.; Jeevan-Raj B.; Netter H.-J. Hepatitis B Virus (HBV) Subviral Particles as Protective Vaccines and Vaccine Platforms. Viruses 2020, 12, 126 10.3390/v12020126. PMC7077199 31973017

[ref41] Doering T. L.; Cummings R. D.; Aebi M., Fungi. In Essentials of Glycobiology, Varki A.; Cummings R. D.; Esko J. D.; Stanley P.; Hart G. W.; Aebi M.; Darvill A. G.; Kinoshita T.; Packer N. H.; Prestegard J. H.; Schnaar R. L.; Seeberger P. H., Eds.; Cold Spring Harbor Laboratory Press Copyright 2015–2017 by The Consortium of Glycobiology Editors, La Jolla, California. All rights reserved: Cold Spring Harbor (NY), 2015; 293–304.

[ref42] Abbott R. K.; Crotty S. Factors in B cell competition and immunodominance. Immunol. Rev. 2020, 296, 120–131. 10.1111/imr.12861. 32483855 PMC7641103

[ref43] Chen W.; Stanley P. Five Lec1 CHO cell mutants have distinct Mgat1 gene mutations that encode truncated N-acetylglucosaminyltransferase I. Glycobiology 2002, 13, 43–50. 10.1093/glycob/cwg003. 12634323

[ref44] Wada Y.; Azadi P.; Costello C. E.; Dell A.; Dwek R. A.; Geyer H.; Geyer R.; Kakehi K.; Karlsson N. G.; Kato K.; Kawasaki N.; Khoo K.-H.; Kim S.; Kondo A.; Lattova E.; Mechref Y.; Miyoshi E.; Nakamura K.; Narimatsu H.; Novotny M. V.; Packer N. H.; Perreault H.; Peter-Katalinić J.; Pohlentz G.; Reinhold V. N.; Rudd P. M.; Suzuki A.; Taniguchi N. Comparison of the methods for profiling glycoprotein glycans—HUPO Human Disease Glycomics/Proteome Initiative multi-institutional study. Glycobiology 2007, 17, 411–422. 10.1093/glycob/cwl086. 17223647

[ref45] Stadlmann J.; Pabst M.; Kolarich D.; Kunert R.; Altmann F. Analysis of immunoglobulin glycosylation by LC-ESI-MS of glycopeptides and oligosaccharides. Proteomics 2008, 8, 2858–2871. 10.1002/pmic.200700968. 18655055

[ref46] Reed L. J.; Muench H. A Simple Method Of Estimating Fifty Per Cent Endpoints12. Am. J. Epidemiol. 1938, 27, 493–497. 10.1093/oxfordjournals.aje.a118408.

