# Supplementary material for: Sialic Acid and Fucose Residues on the SARS-CoV-2 Receptor-Binding Domain Modulate IgG Antibody Reactivity
Source: ACS Infect Dis. 2022 Aug 18;8(9):1883–93. doi: 10.1021/acsinfecdis.2c00155 (PMC9469093; doi:10.1021/acsinfecdis.2c00155)
Supplement: Supplementary file 1 — id2c00155_si_001.pdf [file id2c00155_si_001.pdf]

Supplementary data

Table S1. Percentage distribution between different amounts of antenna. The lower part of the table shows presence of LacDiNAc, degree of fucosylation and degree of sialylation on complex type glycans produced in CHO-S and HEK293F-cell lines.

	N331		N343	
	CHO-S	HEK293F	CHO-S	HEK293F
mono-antennary	4.3	4.8	7.8	2.3
mono-antennary/bi-antennary†		12.1		25.7
bi-antennary	52.2	52.5	68.5	60.4
bi-antennary/tri-antennary†		21.5		8.8
tri-antennary	26.3	5.5	19.5	1.9
tri-antennary/tetra-antennary/repeats†	17.1	3.6	4.3	0.8
LacDiNAc*	0.0	79.7	0.0	70.3
Fucose*	95.9	100.0	91.5	99.1
Sialic acid*	65.0	38.6	47.9	14.1

† Individual fragment ion spectra supporting the presence of either single HexNAc or DiHexNAc were observed for the glycoform of the same glycan composition, making it not always possible to assign an unambiguous number of the antenna structures. For relative quantification purposes, those are presented as dual assignment structures.

* The percentage is calculated in relation to total amount of complex structures.

Table S2. Percentage of structures carrying zero to four fucose groups within the same structure showed for site N331 and N343 produced in CHO-S, HEK293F and Lec3.2.8.1-cells. The lower part of the table shows the fucosylation level within the observed glycoforms.

	N331			N343		
# of Fucose residues	CHO-S	HEK293F	Lec3.2.8.1	CHO-S	HEK293F	Lec3.2.8.1
0	26.8	3.4	68.6	21.6	6.5	93.7
1	73.0	54.8	31.4	78.3	69.9	6.3
2	0.2	36.2	0.0	0.0	21.3	0.0
3	0.0	5.6	0.0	0.0	2.0	0.0
4	0.0	0.1	0.0	0.0	0.3	0.0
Oligomanose*	0.0	0.0	34.1	0.0	0.0	7.9
Paucimannose*	32.8	86.6	60.5	56.5	62.5	0.0
Hybrid*	94.3	100.0	0.0	55.2	60.4	0.0
Complex*	95.9	100.0	100.0	91.5	99.1	0.0

* The percentage is calculated in relation of total amount of all observed glycoforms within the given glycan group, i.e. oligomannose, paucimannose, hybrid and complex. For the percentage distribution of the glycoforms at each site see Table 1.

Table S3. Percentage of detected structures carrying zero to four sialic acids within the same glycan structure.

	N331		N343	
# of Sialic acid residues	CHO-S	HEK293F	CHO-S	HEK293F
0	35.0	61.6	52.1	85.9
1	36.6	32.6	28.3	11.6
2	24.7	5.4	19.2	2.5
3	3.7	0.3	0.4	0.0
4	0.1	0.0	0.0	0.0

The percentage is calculated in relation of total amount of all observed hybrid- and complex-type glycoforms, the unoccupied and all oligomannose glycoforms were excluded from calculation.

Table S4. Percentage of O-linked structures at site T323/S325 and T523 when produced in CHO-S, HEK293F, and Lec3.2.8.1-cells.

	T323/S325			T523		
Glycan composition	CHO-S	HEK293F	Lec3.2.8.1	CHO-S	HEK293F	Lec3.2.8.1
HexNAc1	9.9	8.2	78.1	16.9	20.9	99.3
HexNAc1Hex1	9.9	5.3	21.6	3.6	2.6	0.7
HexNAc2	0.0	0.2	0.0	0.0	0.0	0.0
HexNAc2Hex1	0.0	0.9	0.2	0.0	0.0	0.0
HexNAc1Hex1NeuAc1	65.6	14.3	0.0	38.0	5.7	0.0
HexNAc2Hex2	0.0	1.1	0.0	0.0	0.7	0.0
HexNAc3Hex1	0.0	2.6	0.0	0.0	0.0	0.0
HexNAc2Hex1NeuAc1	0.0	5.3	0.0	0.0	2.4	0.0
HexNAc1Hex1NeuAc2	14.6	36.0	0.0	41.5	55.4	0.0
HexNAc2Hex2NeuAc1	0.0	6.4	0.0	0.0	5.9	0.0
HexNAc3Hex1NeuAc1	0.0	9.9	0.0	0.0	0.0	0.0
HexNAc2Hex2NeuAc2	0.0	9.5	0.0	0.0	6.4	0.0
HexNAc3Hex1NeuAc2	0.0	0.1	0.0	0.0	0.0	0.0

The percentage is calculated based on all detected glycoforms on the sites, the unoccupied peptides were excluded from the calculations. HexNAc = N-acetylhexoseamine, Hex = Hexose, NeuAc = Sialic acid.

Table S5. Characterization of convalescent sera. Neutralization capability determined using a viral CPE assay. Anti-RBD IgG levels as determined using an automated CMIA (values ≥ 50 AU/mL is considered positive). The neutralization capability group is stated as decided by the Viral CPE assay.

Neutralization capability group (viral CPE assay)	Sera Number	Neutralizing titre (viral CPE assay)	Anti-RBD IgG AU/mL
**Non-neutralizing (NT negative)**	1	Neg	107.4
	2	Neg	337.8
	3	Neg	205.7
	4	Neg	148.0
	5	Neg	Neg
	6	Neg	Neg
	7	Neg	Neg
**Weakly neutralizing (NT titre 3-6)**	8	3	Neg
	9	3	Neg
	10	4	Neg
	11	4	Neg
	12	4	Neg
	13	4	67.6
	14	6	Neg
**Highly neutralizing (NT titre 48-96)**	15	48	284.6
	16	64	252.0
	17	64	186.0
	18	96	55.9
	19	96	211.4
	20	96	551.6
	21	96	154.3
	22	96	148.5
	23	96	1131.7
	24	96	532.1

Table S6. Amino acid sequences of the recombinant RBD including a C-terminal His-tag.

**RBD**	RVQPTESIVRFPNITNLCPFGEVFNATRFASVYAWNRKRISNCVADYSVLYNSASFSTFKCYGVSP<br>TKLNDLCFTNVYADSFVIRGDEVRQIAPGQTGKIADYNYKLPDDFTGCVIAWNSNNLDSKVGGN<br>YNYLYRLFRKSNLKPFERDISTEIYQAGSTPCNGVEGFNCYFPLQSYGFQPTNGVGYQPYRVVVL<br>SFELLHAPATVCGPKKSTNLVKNKCVNFHHHHHH

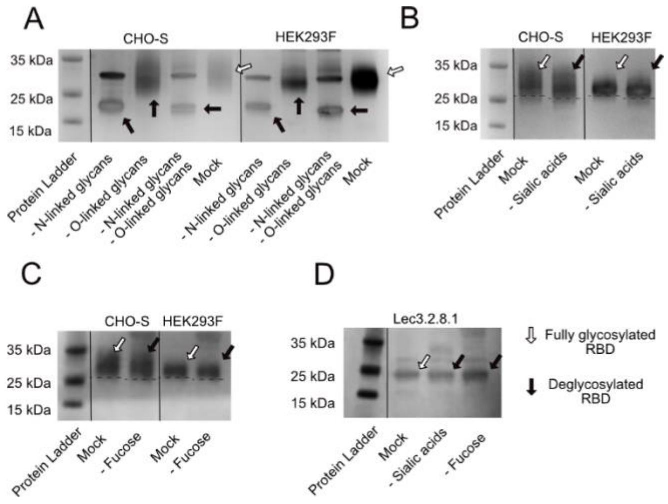

Figure S1. Silver stain of SDS page gel showing band size shift after deglycosylation. A. Band size shift after removal of N-linked, O-linked or both N-linked and O-linked glycans from RBD produced in CHO-S and HEK293F-cells. Open arrows represent fully glycosylated constructs while solid arrows represent the deglycosylated variants. Remaining bands originates from enzymes used in the deglycosylation-reaction. B. Band size shift following sialidase treatment of recombinant RBD produced in CHO-S and HEK293F-cells. C. Band size shift following fucosidase treatment of recombinant RBD produced in CHO-S and HEK293F-cells. D. Band size following sialidase and fucosidase treatment of recombinant RBD produced in Lec3.2.8.1-cells. Data information: The PageRuler™ Prestained Protein ladder is used as size comparison.

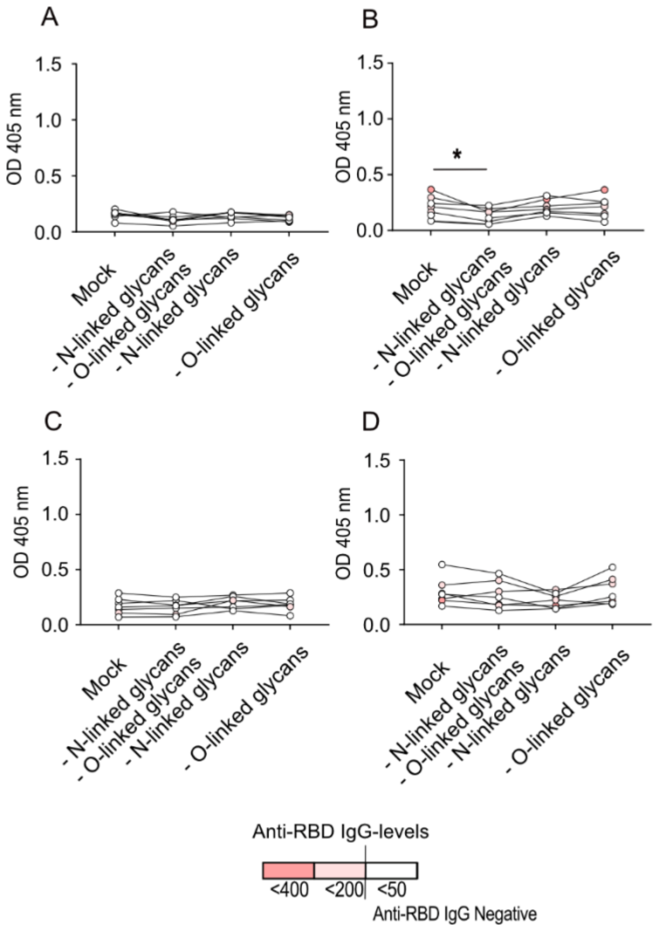

Figure S2. Serum reactivity against the fully glycosylated RBD (mock-treated) and against RBD following removal of N-linked, O-linked or both N-linked and O-linked glycans. A. Antibody reactivity of weakly neutralizing sera (NT titre [illegible], n=7) against RBD produced in CHO-S-cells. B. Antibody reactivity of non-neutralizing sera (NT negative, n=7) against RBD produced in CHO-S-cells. C. Antibody reactivity of weakly neutralizing sera (NT ti[illegible] 3-6. n=7) against RBD produced in HEK293F-cells. D. Antibody reactivity of non-neutralizing sera (NT negative, n=7) against RBD produced in HEK293F-cells. Data information: Dark red colour symbolizes a serum with high levels of anti-RBD IgG, white colour indicates anti-RBD IgG-negative serum (<50 AU/mL). Statisitical analysis was performed with Wilcoxon matched-pair signed rank test. * = $p < 0.05$.

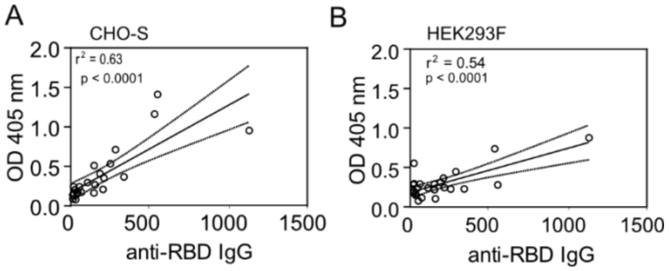

Figure S3. Correlation between anti-RBD IgG titres and OD (405 nm) as measured with ELISA using fully glycosylated RBD. A. RBD produced in CHO-S-cells. B. RBD produced in HEK293F cells. Data information: Pearson correlation coefficient analysis showed a strong correlation ($p < 0.0001$) between high anti-RBD IgG-levels and high OD-value for both CHO-S and HEK293F-produced constructs.

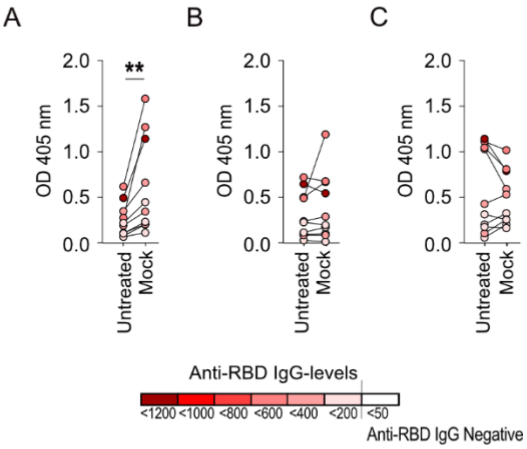

Figure S4. Serum reactivity against untreated and mock-treated RBD. A. Antibody reactivity of highly neutralizing sera (NT tit[illegible]8-96, n=10) against RBD produced in CHO-S-cells. B. Antibody reactivity of highly neutralizing sera (NT titre 48-96, n=10) against RBD produced in HEK293F-cells. C. Antibody reactivity of highly neutralizing sera (NT titre 48-96, n=10) against RBD produced in Lec3.2.8.1-cells. Data information: Dark red colour symbolizes a serum with high levels of anti-RBD IgG, white colour indicates anti-RBD IgG-negative serum (<50 AU/mL). Statisitical analysis was performed with Wilcoxon matched-pair signed rank test. ** = $p < 0.01$.

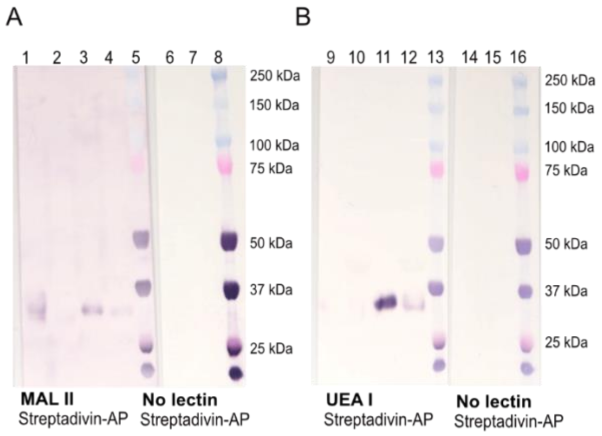

Figure S5. Lectin blots confirming enzymatic removal of sialic acid- and fucose residues from CHO-S- and HEK293F-produced RBD. Lane 1: 2 µg SARS-CoV-2 RBD produced in CHO-S, mock treated, Lane 2: 2 µg SARS-CoV-2 RBD produce[illegible] CHO-S, neuraminidase treated, Lane 3: 2 µg SARS-CoV-2 RBD produced in HEK293F, mock treated, Lane 4: 2 µg SARS-CoV-2 RBD produced in HEK293F, neuraminidase treated, Lane 5: MW Precision Dual Color (Bio-Rad), Lane 6: 2 µg SARS-CoV-2 RBD produced in CHO-S, mock treated. Lane 7: 2 µg SARS-CoV-2 RBD produced in HEK293F, mock treated, Lane 8: MW Precision Dual Color (Bio-Rad), Lane 9: 2 µg SARS-CoV-2 RBD produced in CHO-S, mock treated, Lane 10: 2 µg SARS-CoV-2 RBD produced in CHO-S, fucosidase treated, Lane 11: 2 µg SARS-CoV-2 RBD produced in HEK293F, mock treated, Lane 12: 2 µg SARS-CoV-2 RBD produced in HEK293F, fucosidase treated, Lane 13: MW Precision Dual Color (Bio-Rad), Lane 14: 2 µg SARS-CoV-2 RBD produced in CHO-S, mock treated, Lane 15: 2 µg SARS-CoV-2 RBD produced in HEK293F, mock treated, Lane 16: MW Precision Dual Color (Bio-Rad).
